# Effects of sarpogrelate hydrochloride on peripheral arterial disease: A meta-analysis of randomized controlled trials

**DOI:** 10.1097/MD.0000000000017266

**Published:** 2019-11-15

**Authors:** Yunxin Lu, Jiangmiao Li, Jiayi Xie, Qingliang Yu, Liang Liao

**Affiliations:** Guangxi Medical University.

**Keywords:** 5-HT_2A_ receptor, meta-analysis, peripheral arterial disease, sarpogrelate hydrochloride

## Abstract

**Objective:**

The aim of our study was to assess the efficacy and safety of sarpogrelate hydrochloride by comparing the effects of sarpogrelate with conventional treatment on the improvement of symptoms in PAD patients.

**Methods:**

The search was conducted in PubMed, Embase, Cochrane library database, CNKI, CBM for relevant randomized controlled trials (RCTs) before January 1st, 2019. Inclusion and exclusion of studies, assessment of quality, outcome measures, data extraction and synthesis were completed by two reviewers independently. The meta-analysis was performed with RevMan 5.3.

**Results:**

Totally, 12 eligible RCTs were included in our analysis. Comparing the results of sarpogrelate group and control group, sarpogrelate significantly improved ankle-brachial index (ABI) levels (SMD = 0.05, [95%CI 0.20 to 0.74, *P* = .0005]), dorsalis pedis artery blood flow (MD = 0.16, [95%CI 0.09 to 0.23, *P* < .001]) and pain-free walking distance (PFWD) (MD = 201.86, [95%CI 9.34 to 394.38, *P* = .04]). The pooled analysis showed that a significant decrease in hsCRP (MD = -0.57, [95%CI -1.12 to -0.02, *P* = .04]) and IL-6 (MD = 1.48,[95%CI 0.39 to 2.56, *P* = .008]) was observed in the sarpogrelate treatment.

**Conclusion:**

Sarpogrelate was effective for improving the symptoms of PAD and showed good tolerability without significant adverse events.

## 1 Introduction

Peripheral arterial disease (PAD) was the main manifestation of atherosclerosis.^[1]^ Peripheral artery occlusion can reduce blood flow in limbs to cause rest pain. Globally, PAD had an estimated prevalence of approximately 3% to 10%, increasing to 15% to 20% in people over 70s.^[2,3]^ The symptom tended to be worse and had serious complications, including intermittent claudication, diabetic foot and even critical limb ischemia (CLI).^[4,5]^ Without effective treatment, the risk of amputation and mortality hugely increased.^[6,7]^ Also, there existed unsatisfactory postoperation for CIL patients. The rate of atherosclerotic restenosis was high after the operation. Now, the drugs such as anticoagulants, thrombolytics, antiplatelet drugs were commonly used to relieve symptoms of PAD patients.^[8]^

Sarpogrelate hydrochloride, as a serotonin receptor antagonist, was recommended for improving ischemic changes by inhibiting thrombosis and vasoconstriction in recent years.^[9–11]^ By blocking serotonin receptors, sarpogrelate inhibited platelet aggregation and induced vasodilation as well as reducing vascular endothelial cell damage without affecting normal tissue.^[12,13]^ However, the application of sarpogrelate may lead to a series of adverse reactions, such as liver dysfunction, allergic reactions, and gastrointestinal reactions.^[14]^

Although sarpogrelate had been well promoted, the application of sarpogrelate was controversial because of the poor quality of the literature and the lack of reliable data. It was necessary to accurately assess the efficacy and safety of sarpogrelate. Therefore, we performed meta-analysis by comparing sarpogrelate with conventional therapy for PAD. Furthermore, we also analyzed adverse events of sarpogrelate over the data.

## 2 Methods

### 2.1 Literature search

A systematic literature search was conducted in PubMed, Embase, Cochrane library database, CNKI, CBM. All relevant publications, published before January 1, 2019, were included. The following search terms were used: “sarpogrelate,” “MCI-9042” “Anplag,” “PAD,” “Intermittent claudication,” “Deep vein thrombosis,” “Arteriolar occlusion,” “Skin microvascular lesions,” “Diabetic foot,” “vasculitis angiitis,” “Arteriosclerosis obliterans”. Reference lists of the previous meta-analysis and systematic reviews and primary articles were included. The language was not limited in our study.

### 2.2 Inclusion and exclusion criteria

Eligible Studies should meet the following criteria:

1. Type of studies was randomized controlled trials.
2. Participants were adults with peripheral vascular disease.
3. The intervention in the experimental group was sarpogrelate and one placebo or another conventional drug as a control group.
4. There was no difference in diet between the experimental group and the control group.
5. They had clear outcome indicators.

The criteria for exclusion were as follows:

1. The experimental group used other drugs in addition to sarpogrelate.
2. Participants had other serious diseases which may affect the results of the experiment.
3. The follow-up period was less than four months.
4. Animals studies.

### 2.3 Assessment of quality

The following evaluated item in bias analysis of RCTs had been taken into consideration: the allocation method was random or not, studies used blinding and whether they had been destroyed, whether secrecy of distribution plan was perfect, whether the experimental results had selective reports and other bias. The grade of quality was “high”, “low”, or “unclear”.

### 2.4 Outcome measures and data extraction

All data evaluated by 2 authors independently. Data extraction forms, based on experimental design, contained the following information: primary information of study (name of first author, sample size), baseline characteristics of the patients (sex, mean age, country, type of disease), design of study (control group, dose, control drug, follow-up time), risk of bias assessment (blind method). The primary outcome was ABI, DAV, and PFWD. IL-6 and hsCRP were secondary outcomes.

### 2.5 Statistical methods

Meta-analysis was conducted with RevMen5.3, provided by the Cochrane Collaboration. A continuous variable was reported as mean difference (MD) or standardized mean difference (SMD). The Cochran's Q-statistic and *I* tests were used to evaluated heterogeneity. We used a random effect model when heterogeneity existed (I^2^ values ≥ 50%). On the contrary, a fixed effect model performed when heterogeneity analysis results showed low risk (I^2^ values <50% or *P* value ≤ .10).

### 2.6 Ethics statement

All analyses were based on previous published studies, thus no ethical approval and patient consent are required.

## 3 Results

### 3.1 Literature search results

**Figure 1:**
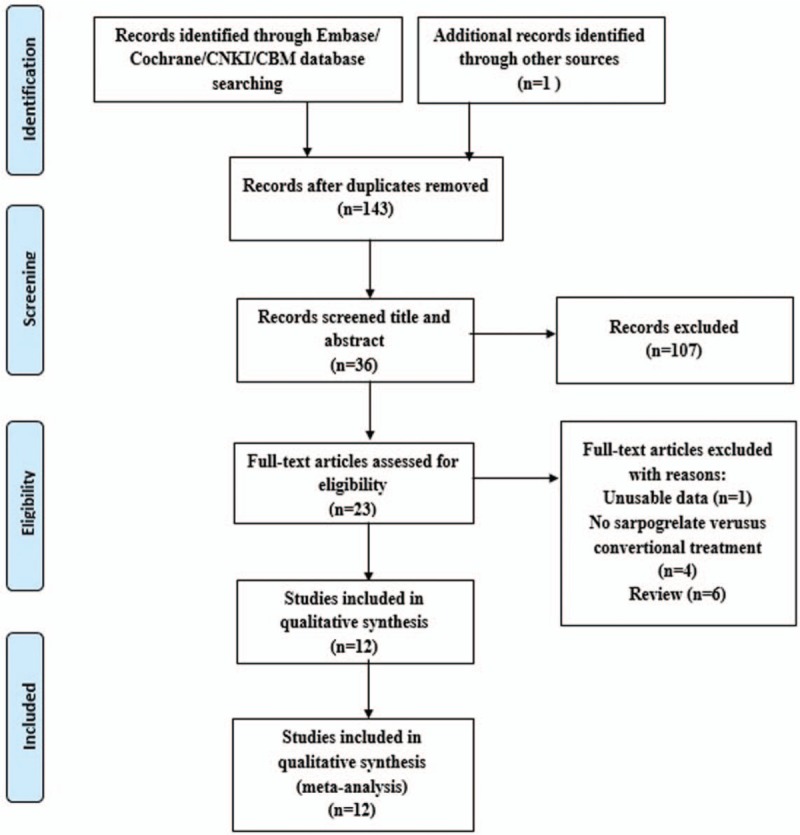
Process of literature search.

A total of 209 possibly relevant articles were identified and reviewed. This flow chart in Figure 1 showed the whole process of literature filtering. Sixty-six studies were excluded due to duplicates and 107 were eliminated after reviewing the titles and abstracts. Of the study screening, the full text of 23 studies was assessed and 12 RCTs met the inclusion criteria (Fig. 1). Eventually, 12 RCTs^[15–26]^ were included in our meta-analysis (Table 1).

**Table 1:**
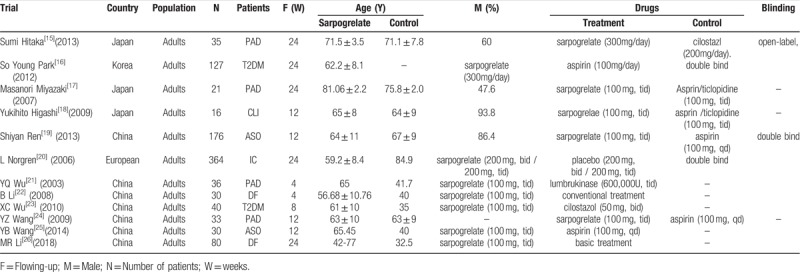
Main characteristics of randomized controlled trials included in the meta-analysis.

### 3.2 Characteristics of included trails

The patient characteristics and experimental design features in each article were shown in Table 1. Twelve RCTs included 988 cases. Seven trails were from China. Three trails were from Japan, one was from Korea and the other one trail was from Europe. The drug comparison was sarpogrelate and conventional treatment (aspirin, cilostazol, ticlopidine, lumbrokinase, or basic treatment).

### 3.3 Meta-analysis results

**Figure 2:**
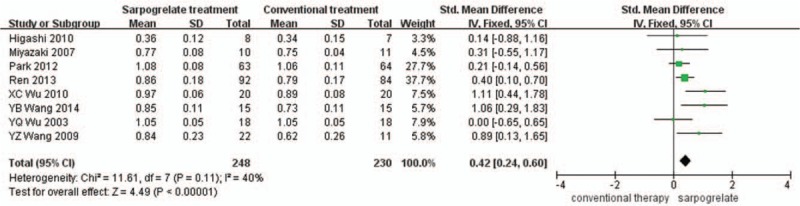
Comparison of the effects of sarpogrelate and conventional treatment on ankle-brachial index.

**Figure 3:**
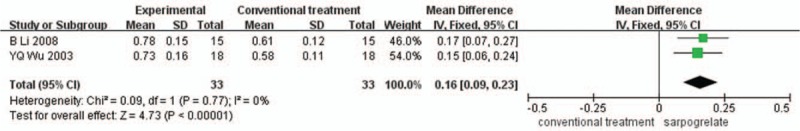
Comparison of the effects sarpogrelate and conventional treatment on dorsalis pedis artery blood flow.

**Figure 4:**
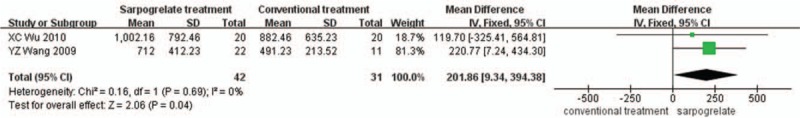
Comparison of the effects sarpogrelate and conventional treatment on pain-free walking distance.

**Figure 5:**
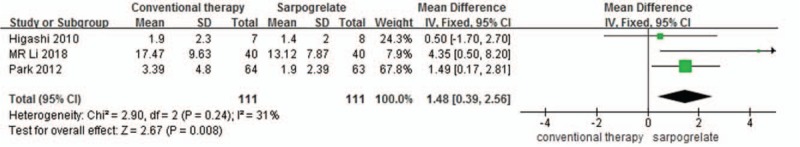
Comparison of the effects sarpogrelate and conventional treatment on Interleukin-6.

**Figure 6:**
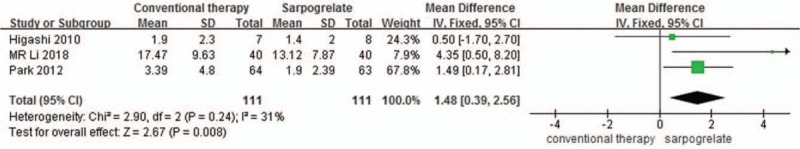
Comparison of the effects sarpogrelate and conventional treatment on hypersensitive C-reactive protein.

**Figure 7:**
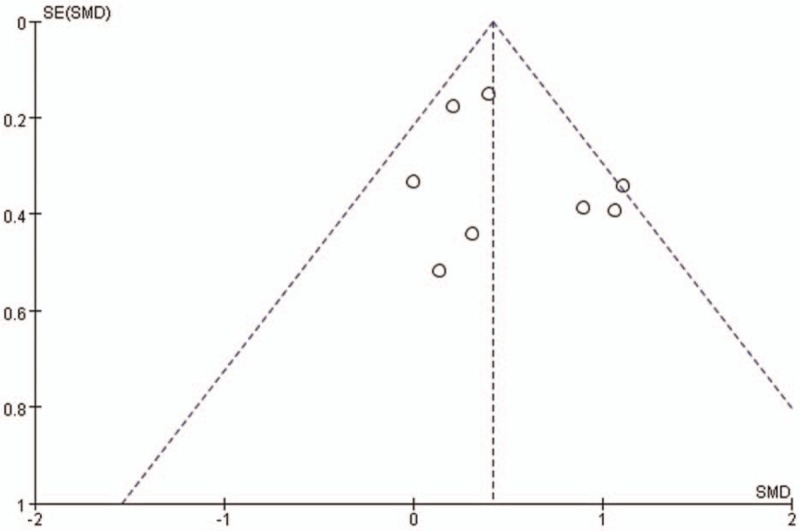
Funnel plot: assessment of publication bias.

Comparison of ABI between the sarpogrelate treatment (Experimental group) and Conventional treatment (control group) showed a statistical heterogeneity between the Eight studies (I^2^ = 31%). A random effect model was used, and the result showed that ABI of sarpogrelate group was higher than the control group (SMD = 0.05, 95%CI 0.01 to 0.08, *P* = .008 Fig. 2). And sarpogrelate treatment increased the dorsalis pedis artery blood flow (I^2^ = 0%, MD = 0.16, 95%CI 0.09 to 0.23, *P* < .001 Fig. 3). In addition, sarpogrelate showed a significant effect on PFWD (MD = 201.86, 95%CI 9.34 to 394.38, *P* = .04, Fig. 4). hsCRP and IL-6 were reported by 3 studies, and there was minimal heterogeneity (hsCRP: I^2^ = 0%, IL-6: I^2^ = 31%). The pooled analysis showed that a significant decrease in hsCRP and IL-6 was observed in sarpogrelate group (IL-6: MD = −1.48, 95%CI −2.56 to −0.392, *P* = .008 Fig. 5; hsCRP: MD = −0.57, 95%CI −1.12 to −0.02, *P* = .04 Fig. 6). The funnel plot (Fig. 7) were mostly symmetrical. There was insufficient evidence of publication bias among the included studies in the meta-analysis of sarpogrelate on PAD.

### 3.4 Risk of bias

**Figure 8:**
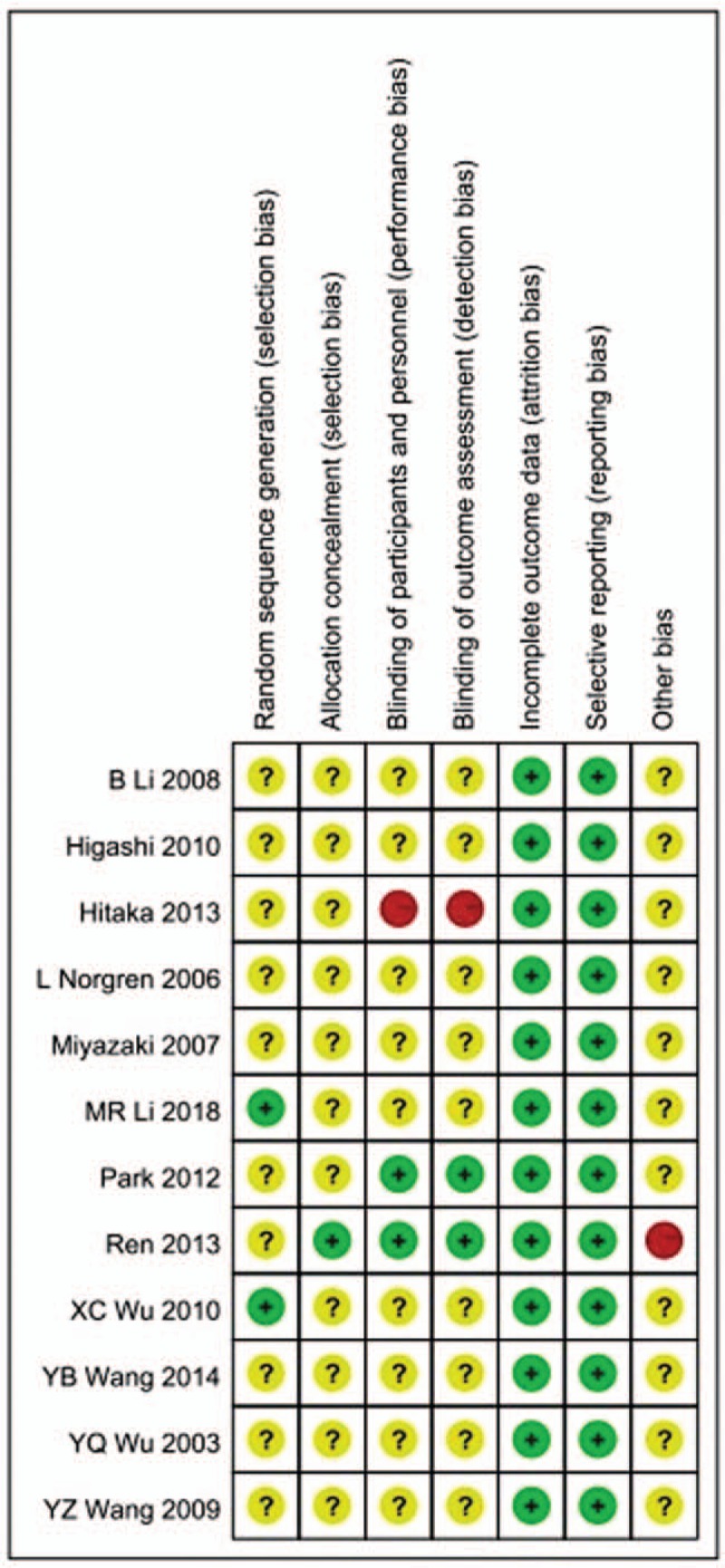
Risk of bias 12 included studies.

As shown in Figure 8, in the 12 included studies, only one with high risk. Two provided high-quality evidence and eight were unclear. Hence, the overall quality of the included RCTs was moderate.

### 3.5 Adverse events

Five of included studies reported the side effect of oral sarpogrelate at an effective dose (Park, L Norgren, BY Wang, YZ Wang, YQ Wu). The following adverse events were included: gastrointestinal reaction, increase of liver enzymes, change of blood pressure and rash. The most frequent adverse event was gastrointestinal reaction. These symptoms had improved after treatment or untreatment.

## 4 Discussion

This meta-analysis aims to assess the efficacy and safety of sarpogrelate hydrochloride compared with conventional treatment. The finding from this study demonstrated that sarpogrelate performed better on primary endpoints than conventional treatment. Sarpogrelate was associated with a significant increase in ABI [SMD = 0.42, 95%CI (0.24, 0.60)], dorsalis pedis artery blood flow [MD = 0.16, 95%CI (0.09, 0.23)] and PFWD [MD = 201.86, 95%CI (9.34, 394.38)]. In addition, sarpogrelate could decrease in IL-6 [MD = 1.48, 95%CI (0.39, 2.56)] and hsCRP [MD = 0.57, 95%CI (0.02, 1.12)].

In our study, sarpogrelate, as a selective 5-HT_2A_ receptor antagonist, could effectively increase ABI, just like the results of Umrani DN and Chen YX.^[27,28]^ Although the sample size in 2 included studies^[21,22]^ was small, it also showed the positive effect of sarpogrelate on dorsalis pedis artery blood flow. Moreover, sarpogrelate could greatly lengthen PFWD of PAD patients compared with conventional treatment. Hiroshi Matsuo suggested that the patient's walking ability was improved after oral administration of sarpogrelate.^[29]^ For PAD patients, peripheral ischemia may be related to the following mechanism: thrombosis, vasoconstriction and smooth muscle cells proliferation. All of them mainly caused by 5-HT_2A_ receptor activation.^[30–34]^ First, endothelial cell damage led to the release of thromboxane A2 and 5-HT, inducing more platelet aggregation. Sarpogrelate slowed down thrombosis by inhibiting the release of 5-HT.^[35]^ Second, vascular dysfunction occurred in PAD patients, leading to insufficient blood supply.^[36]^ Sarpogrelate improved vascular function and suppressed vasoconstriction, which was consistent with the results of Masanori Miyazaki.^[17]^ Third, inhibition of smooth muscle cell proliferation may be associated with sarpogrelate suppression of cell G1 division.^[37]^ In the study of Gao Wei,^[38]^ just as our results, sarpogrelate had a great efficacy on PAD. In contrast, Soga et al were doubtful about the efficacy of sarpogrelate.^[14]^ Due to the small number of study samples and many uncertainties (no clear patient compliance, no clear criteria for inclusion and most patients in serious condition), the proposition of sarpogrelate remained to be investigated.

This meta-analysis also suggested that the IL-6 and hsCRP was in a downward trend in sarpogrelate group. IL-6 and hsCRP, providing molecular markers of the potential severity of atherosclerosis, were known to be strongly associated with PAD.^[39]^ It was reported that 5-HT bound to 5-HT_2A_ receptor and promoted IL-6 synthesis in vascular smooth muscle cells, leading to vasculitis in the development of atherosclerosis.^[12,40]^ Furthermore, Ridker had indicated that CRP might be associated with vascular risk because cytokines such as IL-6 promoted leukocyte adhesion and stimulated vascular endothelial cells to produce CRP.^[41]^ CRP may have a pro-coagulant effect and played an important role in the formation of thrombus.^[41]^ It had been reported that sarpogrelate could decrease serum levels of IL-6 and hsCRP as well as relieving the inflammation of vascular by blocking 5-HT_2A_ receptor.^[12,40]^ Reduced inflammation delayed the progression of atherosclerosis and improved the symptoms of vascular occlusion in patients. In 2 included studies, the serum of cholesterol decreased in sarpogrelate group. It was reported that sarpogrelate may be recommended for preventing atherosclerosis by decreasing serum cholesterol.^[31,32,42]^

There were 3 RCTs including diabetic patients with peripheral vascular disease.^[16,23,26]^ These studies confirmed that sarpogrelate was effective for the therapy of diabetes-related peripheral vascular complications. The result of previous study was consistent with these three RCTs.^[43]^ In above these RCTs, the same basic treatment was performed on T2DM patients in both the experimental and control groups, which had no significant effects on PAD patients. Therefore, sarpogrelate should be taken into consideration in the prevention of diabetes-related peripheral circulatory disturbances.

As far as the safety assessment of sarpogrelate, there were 5 included studies with 590 cases reporting adverse events but not serious.^[16,20,21,24,25]^ With relevant treatment, related symptoms can get relieved. Additionally, Doggrell also suggested that sarpogrelate had seemed to be involved in no serious adverse events and was well-tolerated.^[35]^

Our paper included 12 RCTs with 988 cases and was the latest meta-analysis to evaluate the efficacy and safety of sarpogrelate. We also focused on the effect of sarpogrelate on proinflammatory cytokine. Taken together, this study showed a further understanding of sarpogrelate for PAD. However, this study still had some limitation. The sample size of the included studies was small. Disease subspecies, control group administration and indicators were not exactly uniform. There existed bias in part of eligible articles. Blinding was not used in most included studies, and this may have an impact on the results.

## 5 Conclusion

In conclusion, this study demonstrated that sarpogrelate was effective to improve the symptoms of PAD. Additionally, sarpogrelate showed good tolerability without serious adverse events. Based on the present studies, further investigations need to be conducted to confirm the effectiveness and safety of sarpogrelate.

## Author contributions

**Data curation:** Yunxin Lu, Jiangmiao Li.

**Methodology:** Yunxin Lu, Jiangmiao Li.

**Project administration:** Liang Liao.

**Software:** Yunxin Lu.

**Visualization:** Jiangmiao Li.

**Writing – original draft:** Yunxin Lu, Jiangmiao Li, Jiayi Xie, Qingliang Yu.

**Writing – review & editing:** Liang Liao.
